# Cardiolipin as a Signaling Hub: Evolutionary Conservation and Programmable Platforms Coupling Mitochondrial Stress to Cell Fate

**DOI:** 10.3390/ijms27156868

**Published:** 2026-07-31

**Authors:** Patrice X. Petit

**Affiliations:** Paris Institute for Neuroscience (SSPIN), Centre National de la Recherche Scientifique, CNRS UMR 8003, Université Paris-Cité, Campus des Saint-Germain, 46 rue des Saints-Pères, 75006 Paris, France; patrice.petit@inserm.fr

**Keywords:** cardiolipin, mitochondria, apoptosis, mitophagy, caspase-8, Bid, NLRP3, signaling hub, evolutionary conservation, Barth syndrome

## Abstract

Cardiolipin (CL), a dimeric phospholipid with four acyl chains and a small polar head group, is one of the most striking examples of evolutionary continuity in cell biology. Present in the plasma membrane of α-proteobacteria and retained without fundamental modification in the inner mitochondrial membrane (IMM) of every eukaryote examined, CL has persisted across roughly two billion years of evolution, a period over which the mitochondrion shed the great majority of its ancestral genes. This review develops, as an organizing hypothesis rather than an established fact, the proposal that CL acts as a programmable signaling hub: a lipid whose physical chemistry and membrane address allow it to nucleate distinct supramolecular platforms in response to discrete stress signals, each platform coupling a specific mitochondrial state to a defined cell fate outcome. Three CL-dependent platforms are examined, together with a fourth, emerging axis, and the evidence supporting each is explicitly graded. Platform 1, the catalytic peroxidase platform, converts the constitutive CL–cytochrome *c* (cyt *c*) structural complex into an enzymatic reaction under oxidative stress, generating oxidized CL (oxCL) species that contribute to cyt *c* release from the IMM; this platform is the best supported of the four. Platform 2, the receptor-like mitophagy platform, exploits NME4-dependent CL scramblase activity to translocate CL from the IMM to the outer mitochondrial membrane (OMM) surface upon membrane potential dissipation, creating an externalized “eat-me” signal recognized by LC3-II; the evidence here is moderate and largely cell-based. Platform 3, the caspase-8/BID activation platform, is proposed to assemble a CL microdomain scaffold at the OMM that recruits caspase-8, markedly accelerates BID cleavage, and couple extrinsic apoptotic signals to mitochondrial outer membrane permeabilization (MOMP); this model rests substantially on reconstituted systems and requires further validation in intact cells and in vivo. A fourth, still-debated axis links CL externalization to innate immune activation through NLRP3 recruitment, for which alternative membrane-recruitment models exist. The argument advanced here is that the conservation of CL is unlikely to be explained by its structural roles alone, although those roles are themselves sufficient to impose strong selection; disentangling structural from signaling contributions remains an open problem, and the comparative genomic work needed to do so has not yet been performed.

## 1. Introduction

**Figure 1:**
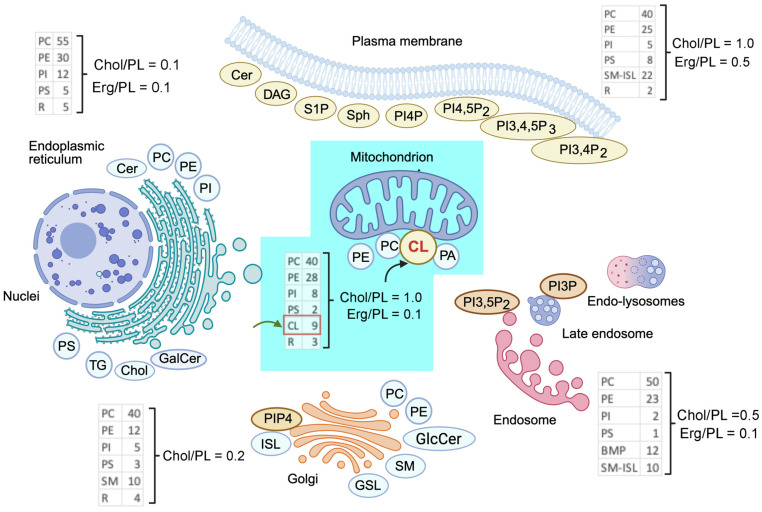
Mitochondrial membrane lipid composition (Patrice X. Petit 2026 Biorender Licence LC29ZMPALR).

Among the many hundreds of glycerophospholipid and sphingolipid species that make up the mammalian lipidome, cardiolipin (CL; 1,3-bis(*sn*-3′-phosphatidyl)-*sn*-glycerol) occupies a singular position. It is the only phospholipid with a dimeric architecture—two phosphatidic acid moieties linked through a central glycerol—and four acyl chains that generate a cone-shaped molecular geometry favoring negative membrane curvature [1,2]. It is also the only lipid whose distribution is essentially confined to a single organelle: in the inner mitochondrial membrane it constitutes 20–25% of total phospholipid, concentrated at cristae rims and contact sites [3,4]. This restriction is evident when the phospholipid composition of mitochondria is compared with that of other cellular membranes (Figure 1).

Phospholipid composition differs markedly between cellular membranes, and mitochondria are compositionally distinctive. Values are expressed as a percentage of total phospholipid (PL) for mammalian (dark blue) and yeast (light blue) membranes; sterol content is given as the molar ratio of cholesterol (mammals) or ergosterol (yeast) to phospholipid. Blue labels indicate the compartment in which each major glycerophospholipid is assembled; red labels those lipids that act in signaling or organelle recognition; with the exception of ceramide, the latter are present well below 1% of total PL. Approximately 45% of mitochondrial phospholipid—chiefly PE, PA and CL—is synthesized by the organelle itself, and CL is essentially restricted to it. Abbreviations, used consistently throughout this review: PC, phosphatidylcholine; PE, phosphatidylethanolamine; PI, phosphatidylinositol; PS, phosphatidylserine; PA, phosphatidic acid; PG, phosphatidylglycerol; CL, cardiolipin; SM, sphingomyelin; Cer, ceramide; GalCer, galactosylceramide; GSL, glycosphingolipid; ISL, inositol sphingolipid; DAG, diacylglycerol; TG, triacylglycerol; BMP, bis(monoacylglycero)phosphate; S1P, sphingosine-1-phosphate; PI4P, PI(4,5)P_2_, PI(3,5)P_2_ and PI(3,4,5)P_3_, the corresponding phosphorylated phosphatidylinositols; ER, endoplasmic reticulum. Adapted from [5] with permission.

**Figure 2:**
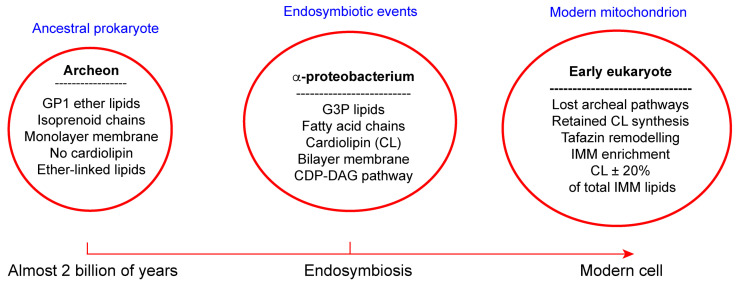
Endosymbiotic origin and continuity of cardiolipin (Adobe creative suite/illustrator).

With only minor structural modification, CL is the same molecule found in the plasma membrane of α-proteobacteria, the group from which mitochondria descended by endosymbiosis approximately two billion years ago [6,7] (Figure 2).

An archaeal host cell and an α-proteobacterial endosymbiont give rise to the early eukaryote, transferring CL from the bacterial plasma membrane to what becomes the inner mitochondrial membrane. In the ancestral prokaryote, CL is enriched in high-curvature domains associated with the respiratory chain; in the modern mitochondrion, it remains confined to the IMM, particularly at cristae rims, where it organizes respiratory supercomplexes (CI–CIII_2_–CIV). The biosynthetic route (PA → CDP-DAG → PGP → PG → CL) and the enzymes that execute it, including cardiolipin synthase (CLS/CRD1), are orthologous across bacteria, fungi, plants and animals. Comparative genomic support for the strength and target of selection acting on these genes is discussed, with its limitations, in Section 2.3.

The textbook account of CL assigns it structural functions: stabilizing respiratory chain supercomplexes (the respirasome, CI–CIII_2_–CIV), maintaining the proton impermeability of the IMM, and providing the negative surface charge that attracts matrix-targeted import sequences [8,9,10]. These roles are real, and it must be stated plainly that they may by themselves be sufficient to account for the conservation of CL and of its biosynthetic pathway. Respiratory efficiency and cristae architecture are fitness-relevant traits, and many essential lipids—phosphatidylethanolamine and phosphatidylglycerol among them—have deeply conserved biosynthetic routes without being regarded as signaling molecules. What the structural account does not obviously explain is why the acyl chain composition of CL should be controlled post-synthetically by a dedicated remodeling enzyme (tafazzin), and why loss of that enzyme produces a systemic disease, Barth syndrome, whose phenotype is not proportionate to the modest respiratory defect it causes [11,12,13]. The question this review addresses is therefore not whether CL has structural roles—it plainly does—but whether those roles account for the whole of the selective pressure acting on it.

The hypothesis explored here is that part of the answer lies in treating CL as a signaling lipid: as a programmable platform molecule that nucleates distinct supramolecular assemblies in response to different mitochondrial stress signals. This reframing generates testable predictions: (i) specific protein–CL interactions should be stimulus-dependent and mechanistically necessary for downstream signaling; (ii) the molecular species of CL should influence the efficiency of each platform; and (iii) conservation of CL should be paralleled by conservation of the enzymes that generate and remodel it. Evidence bearing on all three predictions is reviewed below, in the context of three CL-dependent platforms and one emerging axis. The strength of that evidence is uneven, and Table 1 grades it explicitly; readers should note in advance that prediction (iii) is the weakest of the three, since conservation of biosynthetic machinery is consistent with structural as well as signaling requirements and does not by itself discriminate between them.

## 2. Mitochondrial Phylogeny and the Deep Conservation of Cardiolipin

### 2.1. The Endosymbiotic Origin of CL in Eukaryotes

Phylogenomic analyses of mitochondrial and nuclear-encoded mitochondrial genes consistently place the mitochondrial ancestor within or close to the α-proteobacteria, though the precise sister group remains contested [14,15]. What is not contested is that the Last Eukaryotic Common Ancestor already possessed functional mitochondria, and that every eukaryotic lineage examined—from opisthokonts and plants to excavates such as *Giardia* and *Leishmania*—retains CL or structurally equivalent anionic lipids in mitochondrion-derived compartments [16,17]. Even hydrogenosomes and mitosomes, the most highly derived mitochondrion-related organelles, which have lost the electron transport chain entirely, retain CL biosynthetic machinery in several lineages [18]. This observation is often read as evidence that CL has roles beyond oxidative phosphorylation; the inference is reasonable but not decisive, since these organelles retain membrane protein import and Fe–S cluster assembly, both of which have their own lipid requirements.

The biosynthetic route to CL—phosphatidic acid → CDP-DAG → phosphatidylglycerophosphate (PGP) → phosphatidylglycerol (PG) → CL—is essentially identical in bacteria and eukaryotic mitochondria, with clear orthologous relationships at each enzymatic step [19]. Cardiolipin synthase (CLS1 in mammals, CRD1 in yeast) is a direct homolog of the bacterial enzyme [20]. Importantly, regulatory roles for CL are already documented in bacteria and are not a eukaryotic invention. In *Escherichia coli*, CL converts the replication initiator DnaA from its inactive ADP-bound state to the active ATP-bound form, thereby licensing initiation at *oriC*—a direct, biochemically defined lipid-to-protein regulatory event [21]. CL also accumulates at cell poles and division septa, where it governs the localization and osmosensing behavior of the transporter ProP and of the mechanosensitive channel MscS, coupling membrane physical state to solute transport [22]. These bacterial precedents establish that a CL–protein regulatory interface predates the endosymbiotic event, and they are the closest available answer to the question of what an ancestral CL signaling function would have looked like. What is added in eukaryotes is the remodeling system, absent in bacteria, which introduces a further layer of enzymatic control over the mature lipid species.

### 2.2. Acyl Chain Remodeling: Precision as Evidence of Function

In most mammalian tissues, CL carries four linoleoyl chains (18:2), yielding the species tetralinoleoyl-CL (TLCL) as the predominant molecular form [23]. This is not the product of de novo synthesis—CL is initially synthesized with a heterogeneous acyl chain composition—but of post-synthetic remodeling by tafazzin, a phospholipid transacylase encoded on the X chromosome [11]. The substrate specificity of tafazzin and the tissue-specific TLCL profiles it generates have been conserved from yeast to humans, implying strong selection on CL molecular species rather than CL quantity alone [24].

Barth syndrome, caused by hemizygous loss-of-function mutations in TAZ (the tafazzin gene), provides unambiguous genetic evidence that CL remodeling is clinically essential [11,12]. Affected individuals present with dilated cardiomyopathy, skeletal myopathy, neutropenia, and growth retardation [25]—a phenotype that cannot be explained solely by impaired OXPHOS, since respiratory chain assembly is only modestly affected in Barth syndrome fibroblasts [13]. As discussed below, the acyl chain composition of CL directly determines the efficiency of at least two signaling platforms (Platforms 1 and 3), providing a mechanistic basis for the specificity of disease manifestation [26,27].

### 2.3. Purifying Selection and the Hub Hypothesis

Comparative genomic surveys of CL pathway enzymes across eukaryotic phyla report low dN/dS ratios, indicating that these genes evolve under strong purifying selection [28]. Two caveats must be stated. First, the published estimates are drawn from limited species sampling and were not designed to test hypotheses about CL function; a systematic analysis with dense taxon sampling, site-specific dN/dS estimation and explicit comparison against matched control gene sets has not, to this author’s knowledge, been performed, and the argument would be considerably strengthened if it were. Second, and more fundamentally, purifying selection on a biosynthetic pathway demonstrates only that the product is essential; it cannot by itself identify which function is being selected. Conservation of the CL pathway is therefore equally consistent with a purely structural requirement, and the inference from conservation to signaling is, on its own, circular. The observation that carries more weight is the conservation of the remodeling machinery, since acyl chain identity is a poor candidate determinant of bulk membrane structure but a plausible determinant of protein-binding and peroxidation chemistry—and the platform-specific effects of remodeling defects documented in Section 3.3 and Section 5 provide the mechanistic link that the evolutionary argument alone cannot supply. Even so, the discriminating experiment—selective ablation of a signaling function with structural roles preserved, followed by a fitness measurement—has not been performed in any organism, and the evolutionary case should be read as motivating the hypothesis rather than establishing it.

## 3. Platform 1: The CL–Cytochrome *c* Peroxidase Axis

### 3.1. The Constitutive CL–cyt c Complex

Under homeostatic conditions, 15–20% of the total mitochondrial cytochrome c pool exists in tight association with CL at the IMM [29]. This interaction is electrostatic in origin; the lysine-rich face of cyt *c* (pI ~ 10) binds to the anionic head group of CL and partially occludes the heme crevice, reducing cyt c’s electron transfer activity in its membrane-bound state [30]. This constitutive complex represents a kinetic reservoir that also, critically, positions cyt c adjacent to its enzymatic substrate under pro-apoptotic conditions [31].

### 3.2. Activation of CL-Specific Peroxidase Activity

**Figure 3:**
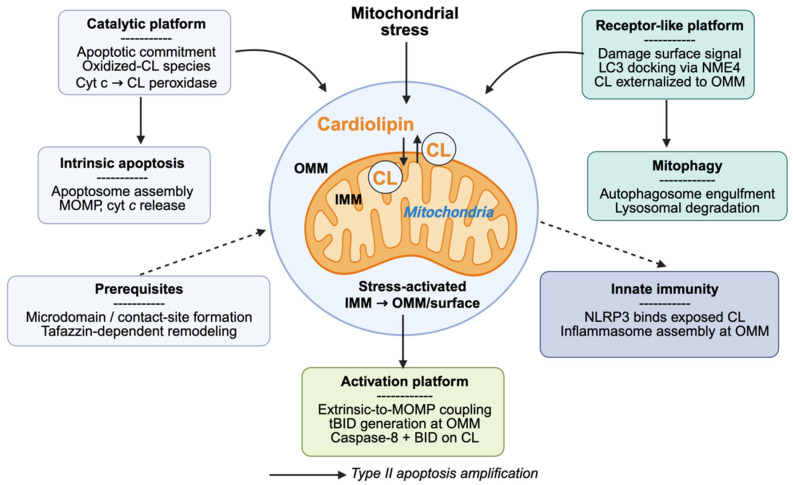
The CL platform modes: a unified overview (Patrice X. Petit 2026 Biorender Licence XL29ZMPVK5/IJMS).

The work of Kagan and colleagues established that, upon oxidative stress, cyt *c* acquires peroxidase activity toward CL [32,33]. The mechanism involves H_2_O_2_-dependent oxidation of the heme iron to compound I/II intermediates that abstract hydrogen from the bis-allylic methylene groups of the polyunsaturated acyl chains of CL (principally 18:2 in TLCL), generating hydroperoxy- and hydroxy-CL species collectively designated oxidized CL (oxCL) [34]. The reaction is enzymatic rather than a random oxidative attack: the oxCL profiles recovered from apoptotic cells are non-random and site-specific, consistent with active-site catalysis [35] (Figure 3). This is the best-supported of the platforms considered here, having been reproduced by independent groups in reconstituted systems, isolated mitochondria and intact cells [36,37].

Two downstream consequences follow. First, accumulation of oxCL weakens the CL–cyt *c* electrostatic interaction, because the modified phosphate environment of oxCL no longer presents an optimal binding surface [38]; this releases cyt *c* into the intermembrane space, where MOMP subsequently permits cytosolic entry and apoptosome assembly. Second, oxCL species are themselves bioactive: they alter IMM curvature, destabilize respiratory supercomplexes, and propagate lipid peroxidation chains that amplify the initial insult [39,40]. It should be emphasized, however, that whether CL peroxidation is *necessary* for cyt *c* release, as opposed to one of several sufficient routes, remains unresolved. Cyt *c* release proceeds in two steps—mobilization of the membrane-bound pool followed by MOMP-dependent translocation—and detachment can be driven by ionic strength, by Ca^2+^, and by tBID/BAX-mediated cristae remodeling in systems where CL oxidation is not detectable [41,42].Conversely, the fraction of the total cyt *c* pool that is CL-bound (15–20%) sets an upper bound on how much of the release this mechanism can account for. The most defensible current reading is that peroxidation is a physiologically important accelerator of cyt *c* mobilization under oxidative conditions, rather than an obligatory gate through which all apoptotic cyt *c* release must pass.

The framework at the center of this review is that CL functions not as a passive membrane component but as a programmable signaling hub operating through distinct, stimulus-coupled platform modes. Three CL-dependent platforms and one emerging axis are shown; each is triggered by a specific upstream stress signal, engages a defined set of protein partners, and produces a discrete cellular output. Note that the innate immune axis (shown in violet) is presented here as an emerging fourth mode whose evidential status is weaker than that of Platforms 1–3 (Table 1). The IMM is drawn in dark gray and the OMM in light gray, with the intermembrane space between them; the blue circular structure at the center of the mitochondrial profile denotes the nucleoid (mitochondrial DNA), included because it is the source of the oxidized mtDNA referred to in Section 6.

Platform 1 (the catalytic peroxidase platform, shown in teal) is activated by mitochondrial reactive oxygen species (ROS) and converts the constitutive CL-cytochrome *c* (cyt *c*) interaction into an enzymatic reaction: cyt *c* acquires CL-specific peroxidase activity, oxidizes CL at polyunsaturated acyl chains, and generates oxidized CL species (oxCL) that weaken CL-cyt *c* binding, ultimately releasing cyt *c* into the intermembrane space (IMS) as an apoptotic initiator.

Platform 2 (the receptor-like mitophagy platform, shown in blue) is activated by mitochondrial membrane potential collapse (Δψm dissipation) and drives NME4-dependent phospholipid scramblase activity that translocates CL from the IMM to the outer mitochondrial membrane (OMM) surface, where externalized CL is recognized by LC3-II on autophagic membranes, selectively targeting the damaged organelle for elimination.

Platform 3 (the caspase activation platform, shown in coral) is activated by extrinsic death receptor signaling and assembles a CL microdomain-based supramolecular complex at the OMM, in which caspase-8 cleaves Bid to generate tBid, which inserts into CL-rich microdomains and drives Bax/Bak oligomerization and mitochondrial outer membrane permeabilization (MOMP).

The dashed arrows indicate proposed cross-talk nodes between platforms and candidate points of therapeutic intervention. These connections are inferences drawn from the individual platform studies rather than direct experimental demonstrations of hierarchy, and are discussed as such in Section 7.

### 3.3. Acyl Chain Composition as a Determinant of Platform Sensitivity

The preference of the cyt *c* peroxidase for polyunsaturated acyl chains explains why TLCL, the product of tafazzin-mediated remodeling, is particularly susceptible. Cells carrying saturated or monounsaturated CL species, as occurs in tafazzin-deficient Barth syndrome cells, show blunted oxCL generation and delayed apoptotic commitment in response to equivalent oxidative insults [26,43]. This provides a mechanistic link between the Barth syndrome phenotype and a specific defect in Platform 1 chemistry, distinct from generic bioenergetic impairment—though it should be noted that tafazzin-deficient cells also differ in monolysocardiolipin content and membrane packing, so the two explanations are not cleanly separable in existing data.

## 4. Platform 2: NME4-Driven CL Externalization and Selective Mitophagy

### 4.1. The Asymmetric Distribution of CL

**Figure 4:**
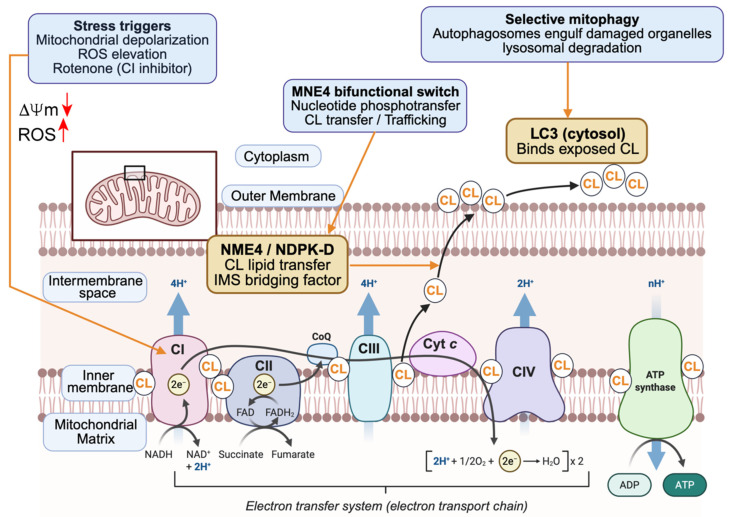
NME4-driven CL externalization and selective mitophagy (Patrice X. Petit 2026 Biorender Licence XX29ZMRGOA/IJMS).

In the resting mitochondrion, CL is asymmetrically distributed, being largely confined to the matrix-facing leaflet of the IMM [44]. The molecular basis of this asymmetry is not settled. No CL-specific flippase has been identified in either mitochondrial membrane, and the ATP-dependent translocases invoked in earlier literature remain hypothetical; the asymmetry is more likely maintained by a combination of the site of synthesis on the matrix leaflet, the very low spontaneous transbilayer diffusion rate of a doubly charged four-chain lipid, and electrostatic retention by the energized membrane [45]. Whatever its origin, loss of membrane potential (Δψm dissipation) is a permissive condition for CL redistribution, and that redistribution is the basis of Platform 2 (Figure 4).

Externalized CL has been proposed as the damage-associated “eat-me” signal that marks a dysfunctional mitochondrion for autophagic engulfment. The figure summarizes the sequence from Δψm dissipation to LC3-II docking. Upper portion: the resting state, with CL confined to the matrix-facing leaflet of the IMM. Central portion: depolarization activates NME4 (nucleoside diphosphate kinase 4), an intermembrane space enzyme that moonlights as a phospholipid scramblase and redistributes CL across the IMM, the IMS and finally to the cytosolic face of the OMM. Right portion: LC3-II on the growing phagophore binds externalized CL directly, without an intervening adaptor. Lower panel: the principal experimental evidence for each step, and the distinction between this route and the PINK1–Parkin ubiquitin-dependent pathway, which operates in parallel and is mechanistically independent.

### 4.2. NME4 as the CL Scramblase

The enzyme responsible for stress-induced CL externalization is NME4 (nucleoside diphosphate kinase 4), a member of the NME/NDPK family that moonlights as a phospholipid scramblase in the intermembrane space [39,46]. Following Δψm dissipation, NME4 catalyzes ATP-independent, bidirectional transfer of CL between leaflets. It is important to be precise about the terminology: NME4 is a scramblase, equilibrating lipids down their concentration gradient without directional preference, and not a flippase; net accumulation of CL at the cytosolic face of the OMM therefore requires a sink on that side. Capture by LC3-II has been proposed to provide such a sink [47], but this remains a plausible model rather than a demonstrated mechanism, and the quantitative contribution of CL synthesis, transfer at contact sites and reverse scrambling has not been measured.

### 4.3. LC3-II Recognizes Externalized CL Directly

LC3-II binds directly to externalized CL without requiring an adaptor protein, a feature that distinguishes this route from PINK1–Parkin-dependent mitophagy [47]. The interaction has been reconstituted with CL-containing liposomes, and independent work has mapped the CL-binding determinants across the human ATG8 family, showing that LC3B, GABARAP and GABARAPL2 differ in their affinity for CL-containing membranes [47,48,49]. In cells, NME4 knockdown abolishes CL surface exposure and blocks CL-dependent mitophagy without affecting PINK1–Parkin-driven mitophagy [39]. The principal limitation of this evidence base is that it rests largely on reconstituted membranes and on knockdown in cultured neuronal cells; genetic loss-of-function evidence in vivo that CL externalization is required for physiological mitophagy is not yet available (Figure 4).

Platform 2 describes a damage-sensing mechanism of considerable economy: the lipid that defines the mitochondrial inner membrane becomes, on translocation to the outer surface, the flag for organelle elimination. In Barth syndrome, impaired CL maturation reduces the density and accessibility of externalized CL, which has been proposed to contribute to the defective mitophagy documented in tafazzin-deficient cardiomyocytes [13,50].

## 5. Platform 3: CL Microdomains as a Caspase-8/BID Activation Scaffold

### 5.1. CL Microdomains at the OMM

CL, despite its predominant IMM localization, is also present at the OMM in discrete, cholesterol-excluding microdomains that are enriched in the pro-apoptotic proteins BAX, BAK, and VDAC [51,52]. These microdomains represent a lateral organization of the OMM distinct from bulk-phase phospholipids and are stabilized by the cone-shaped geometry of CL, which promotes negative curvature and loose packing at microdomain boundaries [53]. Their existence has been demonstrated by detergent-free density gradient fractionation, fluorescence correlation spectroscopy, and super-resolution STED microscopy [52].

### 5.2. Caspase-8 Recruitment and Accelerated BID Cleavage

**Figure 5:**
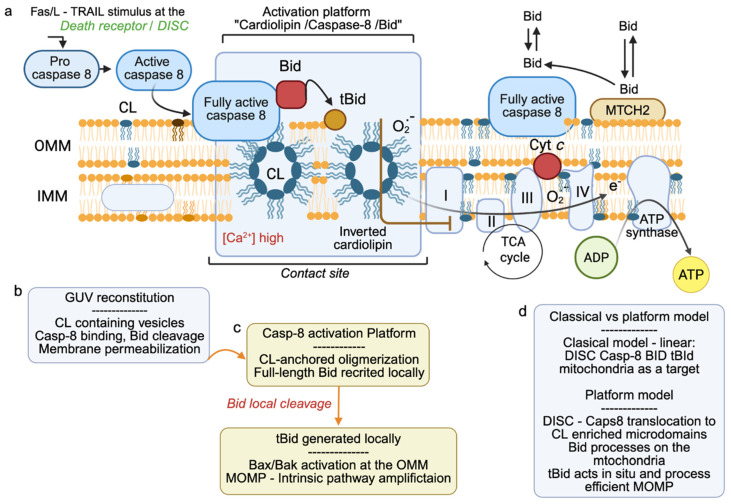
CL microdomains as a caspase-8/BID activation scaffold coupling extrinsic death signals to MOMP (Patrice X. Petit 2026 Biorender Licence WT29ZMRZQN/IJMS).

Upon ligation of death receptors (FAS, DR4/5, TNFR1), activated caspase-8 released from the DISC has been reported to translocate to the OMM surface through direct interaction with CL microdomains [54]. Membrane recruitment raises the local concentration of active caspase-8 and co-concentrates its substrate BID in the same CL-rich environment. In giant unilamellar vesicle (GUV) reconstitutions, the rate of BID cleavage to truncated BID (tBID) is approximately three orders of magnitude greater at CL-containing membranes than in solution [55,56]. This figure should be treated with appropriate caution: it derives from a defined reconstituted system in which lipid composition, protein concentration and the absence of competing substrates are all under experimental control, and in which the effective two-dimensional concentration attainable on a vesicle surface has no direct equivalent in a crowded cytosol. The magnitude of the acceleration in intact cells has not been measured. What the reconstitution does establish robustly is the direction and the mechanism of the effect—surface recruitment accelerates cleavage—rather than its quantitative value in situ. tBID then inserts into the CL microdomain, where it triggers BAX conformational change and oligomerization, culminating in MOMP [57] (Figure 5).

The concept of CL-enriched, raft-like microdomains acting as activating platforms for apoptotic signaling at the mitochondrial surface was introduced by Sorice, Garofalo and colleagues [51,52], building on earlier evidence that mitochondrial structural reorganization is an early event in apoptosis [58]. The specific caspase-8/BID/CL platform described here has since been examined in reconstituted GUVs, isolated mitochondria and intact cells subjected to extrinsic apoptotic stimulation [56,59], and independent support for CL as an assembly surface for apoptotic effectors has come from other groups [37]. The GUV reconstitutions are informative in a specific sense: a minimal system comprising CL-containing membranes, caspase-8, BID and BAX reproduces the sequence from BID cleavage to membrane permeabilization, establishing minimal sufficiency [59]. They do not, however, establish that this is the dominant route in cells, where DISC-proximal cleavage of BID in the cytosol occurs in parallel; the relative flux through the two routes remains undetermined.

The classical model of apoptosis posits a strict separation between the extrinsic pathway (death receptor → caspase-8 activation) and the intrinsic pathway (mitochondrial → MOMP → cyt *c* → apoptosome), connected only through caspase-8 cleavage of BID. This figure presents evidence that CL microdomains at the OMM constitute a physical platform that dramatically accelerates and amplifies this connection, effectively functioning as a signal integration surface at the mitochondrial outer membrane.

The left panel depicts the resting OMM with CL organized into discrete, cholesterol-excluding lipid microdomains (shown as darker patches) that are enriched in BAX, BAK, and VDAC. Upon death receptor ligation (FAS-L, TRAIL, TNF), activated caspase-8 is recruited to the OMM surface. A step that requires direct caspase-8 interaction with CL, where it cleaves BID with approximately 1000-fold greater efficiency than in the cytosol, as shown in the kinetic inset.

The central panel shows tBID insertion into the CL microdomain, where it triggers BAX conformational change and oligomerization, leading to the formation of proteolipidic pores in the OMM and MOMP. A critical mechanistic detail illustrated in the lower inset is that CL acyl chain remodeling by tafazzin determines microdomain fluidity and packing, and therefore the efficiency of caspase-8 recruitment and tBID insertion: Barth syndrome cells with CL remodeling deficiency show blunted Platform 3 responses despite intact upstream death receptor signaling.

The right panel indicates candidate points of pharmacological intervention at this platform. These are conceptual targets rather than validated ones: no compound is currently known to act selectively on CL microdomain assembly or on the caspase-8/CL interface, and the schematic should be read as a statement of where intervention might in principle be attempted, not as a summary of available agents. See Section 8.

### 5.3. The 2025 Extension: Multiple Pathway Entanglements

Two recent studies have extended the Platform 3 model in two directions [60,61]. The first reports that Platform 3 intersects with other regulated cell death pathways—including necroptosis, through RIPK3 association with CL microdomains, and ferroptosis, through GPX4-dependent suppression of CL peroxidation—suggesting a network of CL-dependent pathway entanglements rather than a linear hierarchy [60]. The second maps the binding interfaces within the caspase-8/BID/CL ternary interaction that may in principle be addressed pharmacologically without disrupting the constitutive CL–cyt *c* interaction of Platform 1 [61]. Both studies are recent and, in the case of the necroptosis and ferroptosis connections, rest on a limited body of experimental work; independent replication has not yet been reported, and these proposals should be regarded as provisional. They are included here because they define the questions that most directly test the platform framework, not because they can yet be regarded as settled.

## 6. Emerging Platform: CL and Innate Immune Signaling

Beyond its roles in cell death and quality control, CL has recently emerged as a participant in innate immune signal transduction—a finding that carries particular evolutionary resonance given CL’s bacterial ancestry. The NLRP3 inflammasome, a multimolecular danger-sensing complex that processes pro-IL-1β and pro-IL-18 into mature cytokines and initiates pyroptosis, is activated by mitochondrial signals including externalized CL [62,63].

The mechanistic model for CL-driven NLRP3 activation follows the two-signal scheme of inflammasome biology. A priming signal, typically TLR ligation acting through NF-κB, upregulates NLRP3 and pro-IL-1β. CL externalization to the OMM surface has been proposed to supply the second signal, with NLRP3 docking on exposed CL, recruiting ASC through PYD–PYD interactions and pro-caspase-1 through CARD–CARD contacts to assemble an active complex at the mitochondrial surface [62,63]. Active caspase-1 then processes both cytokine precursors and gasdermin D (GSDMD), whose N-terminal fragment forms plasma membrane pores and drives pyroptosis [64]. This axis is the least secure of those considered in this review, and a competing model should be stated explicitly: NLRP3 has also been shown to be recruited to phosphatidylinositol-4-phosphate on the dispersed *trans*-Golgi network, an interaction that is sufficient for inflammasome assembly and that does not require mitochondrial CL [65]. Whether CL binding is an alternative recruitment route, a contributory one, or an in vitro observation of limited physiological weight is not currently resolved, and direct structural evidence for an NLRP3–CL interface is lacking.

A further immune-relevant connection has been drawn to cGAS–STING signaling: oxidized mitochondrial DNA released from damaged mitochondria activates cGAS, and downstream signaling may be amplified by CL-dependent membrane disruption, linking Platform 1 chemistry to innate DNA sensing [66]. The evolutionary reading is attractive—eukaryotic innate immunity may have co-opted the bacterial signature of CL, treating it as a damage-associated molecular pattern with structural kinship to the pathogen-associated patterns of its α-proteobacterial ancestors—but it should be recognized as an interpretive proposal rather than a tested model.

## 7. Integration: CL as a Decision-Making Interface

**Figure 6:**
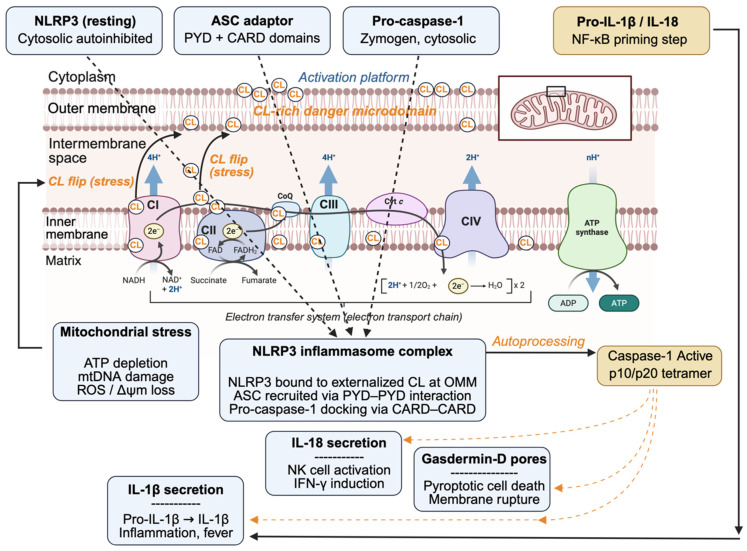
NLRP3/inflammasome/innate immunity axis (Patrice X. Petit 2026 Biorender Licence NT29ZMSRDH/IJMS).

The three platforms and the emerging immune axis are best regarded not as parallel, independent pathways but as an interconnected decision network, with the important caveat that most of the connections between them are inferred from separate studies rather than demonstrated within a single experimental system. Three integration points are worth setting out. First, Platform 1 appears to lie upstream of Platform 2 under mild oxidative stress: peroxidation consumes the polyunsaturated CL species on which the system depends, so that sub-lethal oxCL accumulation depletes the TLCL pool that is the preferred substrate for NME4-mediated scrambling and the preferred ligand for LC3-II. The consequence is reduced efficiency of CL externalization and hence attenuated mitophagy, shifting the threshold between organelle repair and elimination [41]. Second, the magnitude of Platform 1 output may determine whether Platform 3 is engaged: moderate cyt *c* release can initiate apoptosome assembly without MOMP-dependent amplification, whereas maximal release combined with full MOMP requires Platform 3 [39,57]. Third, Platforms 2 and 4 share the NME4-dependent externalization step, which raises the question of what determines whether externalized CL is engaged by LC3-II or by NLRP3 (Figure 6). No regulatory mechanism for this choice has been established. Three non-exclusive possibilities can be framed as testable hypotheses: differential avidity, with LC3-II binding a lower density of exposed CL than NLRP3 requires, making the outcome a function of externalization magnitude; kinetic competition, with the priming state of the cell determining whether assembled NLRP3 is available at the time CL appears; and spatial segregation, with phagophore contact sites and inflammasome nucleation occurring on distinct mitochondrial subdomains. Distinguishing among these would require simultaneous quantification of surface CL density, LC3-II recruitment and NLRP3 assembly in single cells, which has not been performed.

At the lipid level, this integration is governed by CL molecular species. TLCL is the preferred substrate of the cyt *c* peroxidase (Platform 1) and, on current evidence, the preferred ligand for LC3-II docking (Platform 2). The CL compositional defect of Barth syndrome would therefore be expected to degrade the fidelity of both platforms simultaneously, which offers a candidate explanation for a disease phenotype combining altered apoptotic sensitivity, impaired mitophagy and inflammatory dysregulation, a combination not readily reconciled with a purely bioenergetic model [13,26,43,50]. This remains an explanatory hypothesis: it has not been tested by restoring individual platform functions in a tafazzin-deficient background.

The figure is organized in layers. Cytosol (top): the four cytosolic participants before activation—autoinhibited NLRP3, ASC, pro-caspase-1, and the pro-IL-1β/IL-18 generated during NF-κB priming. OMM surface: the CL-rich microdomain on which NLRP3 has been proposed to dock [62]. IMS/IMM: the CL translocation step, shared with the mechanism shown in Figure 4. Inflammasome complex: NLRP3 recruits ASC through PYD–PYD and pro-caspase-1 through CARD–CARD interactions, yielding active caspase-1, with three downstream outputs—IL-1β maturation, IL-18 maturation, and gasdermin-D pore formation leading to pyroptosis. The NLRP3–CL docking step is drawn with a dashed outline to indicate that it remains contested (Section 6).

From a signaling theory perspective, CL behaves as a bifunctional hub molecule: it is constitutively present at the relevant membrane address and constitutively pre-loaded with binding partners (cyt *c*, partially assembled NLRP3 components), but switches from dormant to active states only upon specific stress-triggered modifications (peroxidation, translocation, microdomain reorganization). This architecture, i.e., stimulus-dependent activation of a pre-formed scaffold, resembles the logic of second-messenger systems and kinase cascades more closely than that of a passive structural lipid.

## 8. Therapeutic Implications

Reframing CL as a signaling hub has implications for pharmacology, and it is useful to separate what has been achieved from what remains conceptual. The most significant development is that CL-directed intervention is no longer hypothetical. Elamipretide (SS-31), a mitochondria-targeted tetrapeptide that binds CL and stabilizes the CL–cyt *c* interaction, thereby limiting peroxidase activity and preserving cristae architecture [67], improved knee extensor muscle strength in the TAZPOWER trial in Barth syndrome [68] and received accelerated approval from the U.S. Food and Drug Administration in September 2025 as the first approved mitochondria-targeted therapeutic [69]. Approval was granted on an intermediate clinical endpoint, with continued approval contingent on confirmatory trials, and the trial populations were necessarily very small given a prevalence of roughly 150 patients in the United States; the result should not be over-read. It is nonetheless a proof of principle that the CL–cyt *c* interface is a druggable target in humans, and it is the strongest available support for the argument that CL function is not reducible to bulk membrane structure.

At the preclinical stage, mitochondria-targeted nitroxides such as XJB-5-131 reduce CL peroxidation and limit injury in models of ischemeia–reperfusion and traumatic brain injury [36]. These compounds should be described accurately: they are mitochondrially targeted radical scavengers that reduce oxidative damage to CL among other substrates, and selectivity for oxCL over other oxidized lipids has not been demonstrated. Gene replacement for Barth syndrome, by AAV-mediated *TAZ* delivery, has shown benefit in mouse models but has not entered clinical trials, and faces the delivery, immunogenicity and durability problems common to cardiac and skeletal muscle gene therapy [13].

Beyond these, the remaining proposals in this review are conceptual, and are offered as directions for medicinal chemistry rather than as strategies with existing agents. The caspase-8/CL and BID/CL binding surfaces defined in [61] are candidate interfaces for modulating the extrinsic-to-intrinsic amplification loop, which is of interest both in tumors that evade TRAIL-induced apoptosis and, in the opposite direction, in septic cardiomyopathy and myocardial infarction, where the loop is pathologically over-activated [57]. It should be stated plainly that no compound targeting these interfaces exists, and that “restoring CL microdomain assembly” is at present an aspiration without a defined molecular mechanism: it might in principle mean increasing OMM CL content, shifting acyl chain composition toward TLCL, or stabilizing existing domains against dispersal, and these are different pharmacological problems with different feasibility. Similarly, the observation that Platforms 2 and 4 share the NME4-dependent externalization step suggests that an NME4 modulator might bias the response toward autophagic clearance and away from pyroptosis; no such modulator has been described, NME4 selectivity against other NME/NDPK family members would be a substantial medicinal chemistry challenge, and the therapeutic window is unknown. A useful contrast is provided by NLRP3 itself, for which direct pharmacology already exists: MCC950 acts by locking the NACHT domain in a closed, inactive conformation [70], establishing that conformationally specific inhibition of the inflammasome is achievable and setting a benchmark against which any CL- or NME4-directed approach to the same axis would have to be judged. These ideas are included because the platform framework generates them, not because they are close to translation.

## 9. Limitations, Unresolved Questions and Future Directions

The framework advanced in this review is an organizing hypothesis, and it is worth stating explicitly where it is vulnerable and what would be required to test it.

***The evolutionary argument is not yet decisive***. Conservation of the CL biosynthetic pathway establishes that CL is essential; it does not establish which of its functions is under selection. The dN/dS estimates cited in Section 2.3 were not generated to address this question, and a properly designed comparative genomic analysis—dense taxon sampling, site-specific selection estimates for CLS1/CRD1 and TAZ, and comparison against matched control sets of structural and signaling genes—remains to be performed. Even that would be correlative. The decisive experiment is a separation-of-function allele: a tafazzin or cardiolipin synthase variant that preserves bulk CL content and respiratory supercomplex assembly while selectively degrading a platform function, followed by a fitness measurement in a tractable organism. Yeast, which possesses Platform 1 chemistry but neither death receptors nor an NLRP3 homolog, offers the cleanest system in which to attempt this.

***The platforms are not equally supported, and Table 1 should be read as part of the argument rather than as a summary of it.*** Platform 1 is robust. Platform 2 rests on reconstitution and on knockdown in cultured cells; there is no genetic loss-of-function evidence in vivo that CL externalization is required for physiological mitophagy, and an NME4 conditional knockout with a mitophagy readout would be decisive. Platform 3 is defined chiefly in GUVs; the key missing measurement is the relative flux through membrane-localized versus cytosolic BID cleavage in intact cells, which is technically accessible through FRET-based cleavage reporters combined with CL-binding probes. The innate immune axis is contested, and the outstanding requirement is structural: no NLRP3–CL interface has been resolved, and until one is, the phosphatidylinositol-4-phosphate model [65] remains the better-supported account of NLRP3 membrane recruitment.

***Several specific claims in this review are hypotheses in the guise of mechanisms.*** The proposal that LC3-II capture provides the thermodynamic sink directing NME4-scrambled CL to the OMM surface has not been tested. The determinants of the LC3-II-versus-NLRP3 choice at externalized CL are unknown; the three candidate mechanisms set out in Section 7 are offered as experiments, not as findings. The intersections with necroptosis and ferroptosis described in [60] await independent replication.

***The evidence base is dominated by a small number of experimental systems.*** Reconstituted liposomes and GUVs have been indispensable in defining minimal sufficiency, but they impose defined lipid compositions, omit the protein crowding and curvature heterogeneity of real mitochondrial membranes, and cannot report on flux through competing routes. Much of the cell-based work relies on overexpression or on acute chemical depolarization, neither of which reproduces the graded, chronic stress that is likely to be physiologically relevant. Conversely, the animal data that do exist—principally from tafazzin-deficient models—cannot cleanly separated signaling from bioenergetic contributions, because tafazzin loss perturbs both.

***Contradictory and negative findings deserve more attention than they have received here or in the field.*** The necessity of CL peroxidation for cyt c release is disputed [41,42]; the quantitative significance of the CL-bound cyt c pool is bounded by its size; and reports of CL externalization vary considerably in the magnitude of surface CL detected, in part because the available probes (NAO, MitoCLox, annexin-based reagents) differ in specificity. A systematic re-evaluation of CL surface detection methods would benefit the whole field.

Set against these limitations, the framework earns its place if it generates work that would not otherwise be performed. The most valuable next steps, in the author’s view, are the separation-of-function genetics described above, single-cell simultaneous measurement of surface CL density with LC3-II and NLRP3 recruitment, and structural characterization of the caspase-8/CL and NLRP3/CL interfaces. Each would discriminate between the platform model and its alternatives rather than merely accumulating consistent observations.

## 10. Conclusions

The conservation of cardiolipin across two billion years of eukaryotic evolution is unlikely to be an accident of biosynthetic inertia. This review has argued that it reflects, at least in part, the recruitment and elaboration of CL signaling functions whose antecedents are already visible in bacteria, and which became progressively entangled with eukaryotic cell death, quality control and immunity as the mitochondrion was integrated into the regulatory economy of the cell. The three platforms examined here—catalytic peroxidase, receptor-like mitophagy, and caspase-8/BID activation—together with the emerging innate immune axis, provide a framework that is mechanistically coherent and, more importantly, generates the specific experiments listed in Section 9. The framework should be judged on whether those experiments are worth performing, not on whether the evidence currently in hand compels the interpretation: it does not, and the evidence supporting the four modes is graded accordingly in Table 1. What can be said with confidence is that the inner mitochondrial membrane is not merely a bioenergetic structure, and that cardiolipin is a more active participant in its organization than the textbook account allows.

## Figures and Tables

**Table 1:** The CL-dependent platforms: triggers, components, outputs, evidence grade and inferred evolutionary stage. Evidence grades: *Strong* = reproduced by independent groups across reconstituted, organellar and cellular systems; *Moderate* = consistent cell-based and reconstituted evidence, limited or no in vivo genetic validation; *Developing* = mechanism defined chiefly in reconstituted systems, dominance in cells not established; *Contested* = competing mechanistic models exist, and direct structural evidence is lacking.

Platform	Trigger	CL Event and Key Partners	Cellular Output	Evidence Grade	Inferred Evolutionary Stage
1. Catalytic peroxidase	Mitochondrial ROS/H_2_O_2_	Peroxidation of polyunsaturated acyl chains → oxCL; cyt c, TLCL	cyt c mobilization → apoptosome assembly	Strong	Chemistry available in the α-proteobacterial ancestor; co-opted for apoptosis in the eukaryotic stem lineage, after the origin of the caspase/Apaf-1 machinery
2. Receptor-like mitophagy	Δψm dissipation	NME4-dependent scrambling and CL externalization to the OMM; LC3-II/ATG8 family	Selective autophagic elimination of the organelle	Moderate	Requires the ATG8 conjugation system and is therefore post-LECA in its present form, although CL redistribution on depolarization is probably ancestral
3. Caspase-8/BID scaffold	Death receptor ligation (FAS, DR4/5, TNFR1)	Lateral organization into OMM microdomains; caspase-8, BID/tBID, BAX/BAK, VDAC	MOMP; extrinsic-to-intrinsic amplification	Developing	The latest of the four: death receptors and BID are metazoan innovations, so this platform cannot predate the emergence of animals
4. Innate immune axis (emerging)	CL externalization together with NF-κB priming	Externalized CL as a putative docking surface; NLRP3, ASC, pro-caspase-1, GSDMD	IL-1β and IL-18 Maturation; pyroptosis	Contested	NLRP3 is vertebrate-restricted; the most recent of the modes, and possibly a recognition of CL as an ancestral bacterial signature

## Data Availability

The data presented in this study are available on request from the corresponding author due to (specify the reason for the restriction).
